# S30. UNDERSTANDING THE NATURE OF CHILDHOOD SUSPICIOUSNESS: A QUALITATIVE STUDY

**DOI:** 10.1093/schbul/sby018.817

**Published:** 2018-04-01

**Authors:** Keri Wong

**Affiliations:** University of Cambridge

## Abstract

**Background:**

Paranoia exists on a continuum of severity in adult patient populations and more recently it has been found to exist in children and adolescents in the general population. Childhood paranoia, assessed by the Social Mistrust Scale (SMS), has been found to be related to both internalising and externalising problem behaviours (Wong, Freeman & Hughes, 2014); however, the nature of why children’s suspicions are related to psychosocial functioning remains unexamined. The current qualitative study addresses this gap by following up the original 2014 sample to examining the nature of children’s suspicions using thematic analysis. By giving voice to children and adolescents, I will discuss: 1) children’s definition of trust and mistrust more broadly 2) the common themes generated from interview questions about children’s suspicions in relation to baseline self-reported levels of suspiciousness on the Social Mistrust Scale (SMS) and 3) other developmental psychosocial factors contributing to childhood suspicions. This study is also the first study to address whether or not children’s suspicions are valid, or grounded in reality, using interviewer ratings and child self-report measures of mistrust.

**Methods:**

118 trusting and persistently mistrustful children from the UK (n=40) and Hong Kong (n=78) were matched and followed-up at 6 and 12 months based on their self-reported levels of suspiciousness on the Social Mistrust Scale. Correlations and kappas were conducted to assess the stability and convergent validity between assessments. Thematic analysis was conducted on 95 (80%) randomly selected semi-structured interviews about mistrust. The coding scheme generated from this analysis was further tested on the remaining transcripts for discriminant validity.

**Results:**

Children’s definition of trust was consistent with existing developmental literature. Commonly discussed topics related to mistrust, particularly school mistrust, included (i) experiences of bullying, concerns with popularity and the consequences of being targeted, (ii) emotional worries, anxieties and feelings of hostility, spying, and teasing, and (iii) coping mechanisms that maintained children’s avoidant behaviours. Consistent with the threat anticipation cognitive model of delusions (Freeman et al., 2007), persistently mistrustful children reported frequent peer victimization and hostile attributional bias. Instances of unfounded paranoia were rare but not absent. There was moderate convergent validity between interviewer ratings and the SMS (k=.49, p<.001). The coding scheme discriminated trusting and mistrustful children accurately.

**Discussion:**

Interviews with trusting and persistently mistrustful children are necessary in verifying unfounded childhood suspicions. Complementing self-report measures of suspiciousness, thematic codes from this study have the potential to screen for persistent and strongly held suspicions that may develop into delusions later in life.

